# Design, synthesis, in silico toxicity prediction, molecular docking, and evaluation of novel pyrazole derivatives as potential antiproliferative agents

**DOI:** 10.17179/excli2016-103

**Published:** 2016-03-01

**Authors:** Parameshwar Ravula, Harinadha Babu Vamaraju, Manichandrika Paturi, Narendra Sharath Chandra JN, Swetha Kolli

**Affiliations:** 1 Department of Pharmaceutical Chemistry, Guru Nanak Institutions Technical Campus, School of Pharmacy, Jawaharlal Nehru Technological University, Hyderabad- 501301, India; 2 Medicinal Chemistry Division, G. Pulla Reddy College of Pharmacy, Osmania University, Hyderabad - 500028, India; 3 Department of Pharmaceutical Chemistry, Bojjam Narsimulu Pharmacy College for Woman, Jawaharlal Nehru Technological University, Hyderabad-500028, India

**Keywords:** pyrazolyl tetrazole acetic acids, antiproliferative activity, molecular modeling, VEGFR-2

## Abstract

A new series of pyrazole derivatives were designed by docking into vascular endothelial growth factor receptor-2 (VEGFR-2) kinase active site. The designed compounds were synthesized and evaluated for in vitro antiproliferative activity against HT-29 colon and PC-3 prostate cancer cell lines, and angioinhibitory activity in chorioallantoic membrane (CAM) model. Based on the obtained antiproliferative activity results of in vitro and CAM assay, compounds **4b, 4c, 4f, 5b, 5c **and **5f** were selected, and tested for anticancer activity using in vivo ehrlich ascites carcinoma (EAC) bearing mice. Compound **5c** showed the highest in vitro antiproliferative activity against HT-29 and PC-3 with IC50 values of 6.43 µM and 9.83 µM respectively and comparable to reference drug Doxorubicin. Results of in vivo anticancer activity revealed that compound **5c** showed the highest percentage increase in life span ( %ILS), and mean survival time (MST) with 75.13 % and 32.4 ± 0.53 days respectively. Moreover, compound **5c** demonstrated significant reduction of microvessel density (MVD) in CAM assay. In silico prediction of toxicities, and drug score profiles of designed compounds are promising. A correlation made between the results obtained by antiproliferative study and molecular docking studies suggest that the synthesized compounds may be beneficial as molecular scaffolds for antiproliferative activity.

## Introduction

Cancer is a major chronic disease, known for second leading cause of death worldwide. As per the world cancer report issued by the international agency for research on cancer (IARC), the cancer rates could increase by 50 % to 15 million new cases by 2020. In recent years, small molecules are widely explored as anticancer agents and among them pyrazoles have attracted significant scientific attention, because of their unique structure (Havrylyuk et al., 2009[15]; Liu et al., 2012[19], 2011[21]; Insuasty et al., 2010[17]; Congiu et al., 2010[8]; Omprakash et al., 2013[25]; Liu et al., 2010[20]). On the other hand, tetrazole and its derivatives are reported to possess medicinal properties such as anticancer (Arshad et al., 2014[2]; Gundugola et al., 2010[14]; Saipavankumar et al., 2011[28]; Mahesh et al., 2014[22]), antimicrobial (Rostom et al., 2009[27]) and anti-inflammatory (Bekhit et al., 2004[3]). In the earlier study, we synthesized a series of novel pyrazolyl tetrazoles based on bioisosterism and reported them as cyclooxygenase-2 (COX-2) selective inhibitors (Swetha et al., 2013[33]). COX-2 is an inducible enzyme that is up regulated by a various factors, which include growth factors, cytokines and tumor promoters (Williams et al., 1999[35]). This enzyme is prominently over expressed in cells, which contribute induction of vascular endothelial tubular morphogenesis (Tusjii et al., 1998[34]). Moreover, in recent years, the involvement of COX- 2 enzyme in carcinogenesis was reported by several research groups (Fosslien, 2000[12]; Subbaramaiah et al., 2003[32]). Inspired by this information and continued our research on pyrazoles, herein we started our work with introduction of tetrazole ring on the pyrazole, and allowed ethyl chloroacetate to react with acidic nitrogen of tetrazole to yield desired pyrazolyl tetrazole acetic acids.

Vascular endothelial growth factor (VEGF) and their receptor (VEGFR) are pathologically involved in various processes of angiogenesis (Folkman, 1995[11]; Carmeliet and Jain, 2000[5]) which include endothelial cell (EC) migration, increased vascular permeability, survival and proliferation (Robinson et al., 2000[26]). Among the receptors, VEGFR-2 is more expressed in ECs, and is a key growth factor of various tumor angiogenesis. Consequently, in recent years, this receptor has gained much scientific focus, as target for the design of new anticancer agents (Hiroyuki et al., 2006[16]) and sunitinib (SU-6668), sorafenib (BAY-43-9006) and vandetanib (ZD-6474) are some of clinically used VEGFR-2 inhibitors. Previously, several research groups have been reported pyrazole derivatives as potent VEGFR-2 kinase inhibitors (Miyamoto et al., 2013[24]).

**Figure 1:**
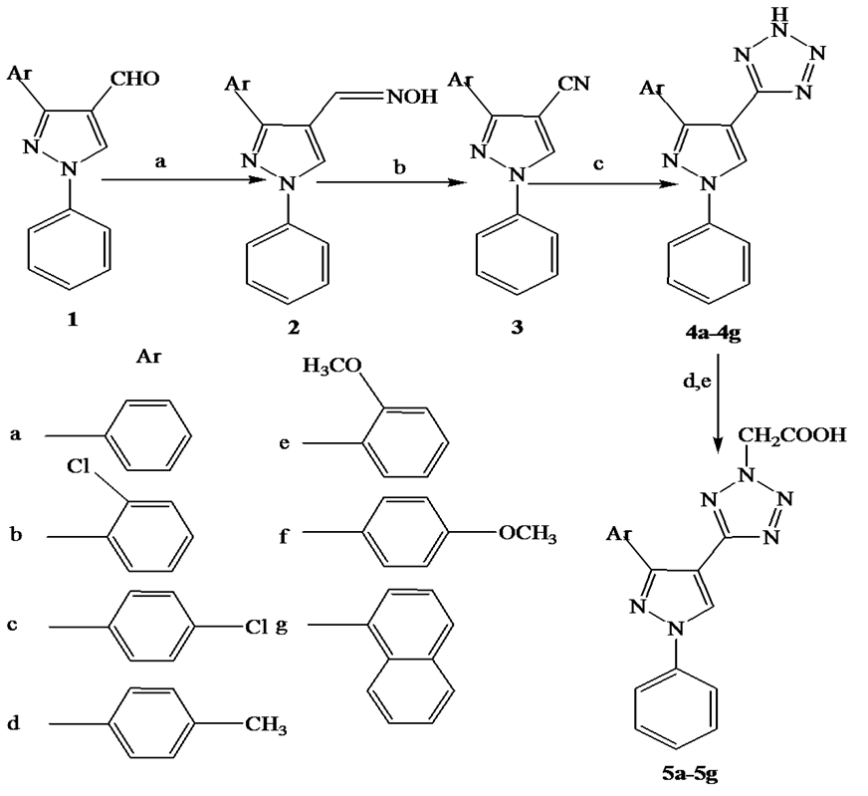
The total synthetic pathway. Reagents and conditions: (a) NH_2_OH.HCl, NaOH, EtOH, reflux, 2h; (b) acetic anhydride, reflux, 1h; (c) NaN_3_, DMF, triethyl ammonium chloride, reflux, 24-48 h; (d) ethyl chloroacetate, K_2_CO_3_, dry acetone, reflux, 4 h; and (e) NaOH, EtOH, reflux, 4h

Based on the above consideration, we designed a new series of pyrazole derivatives using molecular docking studies with the help of software MOE 2008 into the active site of VEGFR-2 kinase. Virtual screening was focused on experimental molecules showing significant interactions as that of marketed drugs used in clinical practice, to the specific active site amino acids of the receptor. Promising molecules in this docking study were synthesized as given in the Figure 1(Fig. 1). The synthesized compounds were screened for *in vitro* antiproliferative activity against two cell lines namely HT-29 colon and PC-3 prostate and *in vivo* using chorioallantoic membrane (CAM) model. Based on the obtained results of these assays, compounds **4b, 4c, 4f, 5b, 5c** and **5f **were tested for *in vivo* anticancer using ehrlich ascites carcinoma (EAC) bearing mice. 

## Materials and Methods

### Chemistry

All reagents were purchased from commercial sources. Melting points were uncorrected and determined in one end open capillary tubes using Analab melting point apparatus. The IR spectra were recorded on Shimadzu FTIR 8400S spectrophotometer and expressed in wave numbers (cm^-1^), using 1 % potassium bromide discs. ^1^H NMR spectra were recorded on Varian 400 MHz spectrometer using DMSO-d_6_ as solvent and tetramethylsilane as an internal standard. The ^13^C NMR spectra of the synthesized derivatives were recorded on Varian Gemini 100 MHz spectrophotometer and mass spectra were recorded on Agilent 6430 triple quadruple LC-MS system. TLC was performed using E.Merck 0.25 mm silica gel plates and visualization of spots was accomplished with UV light (256 nm). 

#### General procedure for the synthesis of 1-phenyl-3-aryl-1H-pyrazole-4-carbonitriles (3)

To a solution of compound **1** (10 mmol) in 20 mL ethanol, was added hydroxylamine hydrochloride (20 mmol), sodium hydroxide (20 mmol) and heated under reflux for 2 h. The reaction mixture was cooled, added to 100 mL of water and neutralized with 0.1N hydrochloric acid. The separated solid was filtered and recrystallized from 90 % ethanol to give compound **2**.

A mixture of compound** 2 **(10 mmol) and freshly distilled acetic anhydride (100 mmol) was heated strongly at 140-150 °C for 1 h to give compound **3**. The reaction mixture was cooled to room temperature and added to crushed ice with stirring. The solid separated was filtered and recrystallized from 90 % ethanol.

#### General procedure for the synthesis of 5-(1-phenyl-3-aryl-1H-pyrazol-4-yl)-2H-tetrazoles(4a-4g)

A mixture of pyrazole nitrile **3 **(10 mmol), sodium azide (20 mmol), and triethyl ammonium chloride (20 mmol) was taken in 15 mL of DMF and refluxed for 24-48 h. The completion of reaction was monitored by TLC. The reaction mixture was cooled, added to 50 mL of water and acidified with 0.1N hydrochloric acid. The separated solid was filtered and recrystallized from chloroform-ethanol mixture to give compound **4.** Physical and spectroscopic data of compounds **4a-4g** have been reported elsewhere (Swetha et al., 2013[33]).

#### General procedure for the synthesis of [5-(1-phenyl-3-aryl-1H-pyrazol-4-yl)-2H-tetrazol-2-yl] acetic acids (5a-5g)

To a mixture of compound **4** (10 mmol) and anhydrous potassium carbonate (10 mmol) in 20 mL of dry acetone, was added ethyl chloroacetate (12 mmol) and refluxed for 4 h. The solvent was removed by evaporation under reduced pressure using a Rotary evaporator and the solid residue obtained was recrystallized from 90 % ethanol to give pyrazolyl tetrazole ester.

A mixture of above ester (10 mmol) and 25 % aqueous sodium hydroxide (5 mL) was taken in 90 % ethanol and refluxed for 4 h. The reaction mixture was cooled, added to 50 mL of water and neutralized with dilute hydrochloric acid. The solid separated was filtered and recrystallized from 90 % ethanol to give compound **5**.

#### 2-(5-(1, 3-diphenyl-1H-pyrazol-4-yl)-2H-tetrazol-2-yl) acetic acid (5a)

Yellow crystals, yield 60 %, mp 220-222 °C. IR (KBr, cm^-1^): 3456 (OH), 3004 (CH aromatic), 1731 (C=O), 1596 (C=N). ^1^H NMR (DMSO-*d*_6_, 400 MHz, δ ppm): 10.61 (s, 1H, COOH), 9.22 (s, 1H, pyrazole), 8.02 (d, 2Ar-H, *J*=7.82 Hz), 7.76 (m, 2Ar-H), 7.57 (t, 2Ar-H, *J*=8.02 Hz), 7.42 (m, 4Ar-H), 5.82 (s, 2H, C*H_2_*COOH). ^13^C NMR (DMSO-*d*_6_, 100 MHz, δ ppm): 166.2 (COOH), 156.8 (tetrazole carbon), 137.9, 134.5, 129.3, 128.8, 127.1, 126.9, 126.5, 125.4, 124.1, 119.4, 104.3, 47.4 (CH_2_). MS (m/z): 346 (M^+^). 

#### 2-(5-(3-(2-chlorophenyl)-1-phenyl-1H-pyrazol-4-yl)-2H-tetrazol-2-yl) acetic acid (5b)

White powder, yield 67 %, mp 196-198 °C. IR (KBr, cm^-1^): 3457 (OH), 3080 (CH aromatic), 1723 (C=O), 1598 (C=N), 754 (C-Cl). ^1^H NMR (DMSO-*d*_6_, 400 MHz, δ ppm): 10.92 (s, 1H, COOH), 9.30 (s, 1H, pyrazole), 7.96 (d, 2Ar-H, *J *= 8.21 Hz), 7.42 (m, 4Ar-H), 7.51 (d, 2Ar-H, *J *= 8.25 Hz), 7.53 (t, 1Ar-H, J = 7.42 Hz), 5.52 (s, 2H, C*H_2_*COOH). ^13^C NMR (DMSO-d_6_, 100 MHz, δ ppm): 165.8 (COOH), 157.7 (tetrazole carbon), 139.5, 134.1, 130.1, 129.4, 128.2, 127.8, 126.9, 126.1, 125.9, 124.5, 123.8, 119.6, 105.5, 49.5 (CH_2_). MS (m/z): 381 (MH^+^). 

#### 2-(5-(3-(4-chlorophenyl)-1-phenyl-1H-pyrazol-4-yl)-2H-tetrazol-2-yl) acetic acid (5c)

White powder, yield 66 %, mp 195-197 °C. IR (KBr, cm^-1^): 3448 (OH), 3124 (CH aromatic), 1724 (C=O), 1596 (C=N), 759 (C-Cl). ^1^H NMR (DMSO-*d*_6_, 400 MHz, δ ppm): 10.50 (s, 1H, COOH), 9.12 (s, 1H, pyrazole), 7.95 (d, 2Ar-H, *J *= 8.21 Hz), 7.62 (m, 4Ar-H), 7.54 (d, 2Ar-H, *J *= 8.21 Hz), 7.45 (t, 1Ar-H, J = 7.43 Hz), 5.50 (s, 2H, C*H_2_*COOH). ^13^C NMR (DMSO-d_6_, 100 MHz, δ ppm): 167.2 (COOH), 157.5 (tetrazole carbon), 138.5, 133.5, 129.5, 129.0, 128.2, 127.8, 127.2, 126.8, 126.1, 118.2, 103.5, 48.5 (CH_2_). MS (m/z): 381 (MH^+^). 

#### 2-(5-(3-(4-methylphenyl)-1-phenyl-1H-pyrazol-4-yl)-2H-tetrazol-2-yl) acetic acid (5d)

Light yellow powder, yield 65 %, mp 210-212 °C. IR (KBr, cm^-1^): 3447 (OH), 3126 (CH aromatic), 2920 (CH aliphatic), 1735 (C=O), 1596 (C=N). ^1^H NMR (DMSO-*d*_6_, 400 MHz, δ ppm): 11.03 (s, 1H, COOH), 9.07 (s, 1H, pyrazole), 7.95 (d, 2Ar-H, *J *= 8.1 Hz), 7.61-7.57 (m, 4Ar-H), 7.49 (d, 2Ar-H, *J *= 8.42 Hz), 7.44 (t, 1Ar-H, *J *=7.20 Hz), 5.52 (s, 2H, C*H_2_*COOH), 2.50 (s, 3H, CH_3_). ^13^C NMR (DMSO-d_6_, 100MHz, δ ppm): 166.8 (COOH), 156.5 (tetrazole carbon), 136.4, 133.4, 132.4, 128.9, 128.6, 127.8, 127.2, 126.9, 125.7, 119.5, 105.6, 48.8 (CH_2_), 22.7 (CH_3_). MS (m/z): 360 (M^+^). 

#### 2-(5-(3-(2-methoxyphenyl)-1-phenyl-1H-pyrazol-4-yl)-2H-tetrazol-2-yl) acetic acid (5e)

Cream powder, yield 68 %, mp 184-186 °C. IR (KBr, cm^-1^): 3417 (OH), 3127 (CH aromatic), 2845 (CH aliphatic), 1740 (C=O), 1543 (C=N), 1027 (C-O). ^1^H NMR (DMSO-*d*_6_, 400 MHz, δ ppm): 10.54 (s, 1H, COOH), 9.24 (s,1H, pyrazole), 8.20 (d, 2Ar-H, *J *= 7.86 Hz ), 7.82 (d, 2Ar-H, *J *= 8.98 Hz), 7.65(t, 2Ar-H, *J *= 8.06 Hz), 7.46 (t, 1Ar-H, *J *= 7.43 Hz), 7.23 (d, 2Ar-H, *J *= 8.61 Hz), 5.64 (s, 2H, C*H_2_*COOH), 3.81 (s, 3H, OCH_3_). ^13^C NMR (DMSO-d_6_, 100 MHz, δ ppm): 167.5 (COOH), 158.8 (tetrazole carbon), 148.9, 139.2, 134.8, 129.9, 128.7, 127.9, 127.4, 124.9, 119.3, 112.3, 120.3, 107.8, 53.4 (CH_2_), 52.6 (OCH_3_). MS (m/z): 376 (M^+^). 

#### 2-(5-(3-(4-methoxyphenyl)-1-phenyl-1H-pyrazol-4-yl)-2H-tetrazol-2-yl) acetic acid (5f)

Cream powder, yield 68 %, mp 185-187 °C. IR (KBr, cm^-1^): 3417 (OH), 3128 (CH aromatic), 2842 (CH aliphatic), 1739 (C=O), 1593 (C=N), 1026 (C-O). ^1^H NMR (DMSO-*d*_6_, 400 MHz, δ ppm): 10.43 (s, 1H, COOH), 9.25 (s, 1H, pyrazole), 8.13 (d, 2Ar-H, *J *= 7.85 Hz ), 7.82 (d, 2Ar-H, *J *= 8.99 Hz), 7.63 (t, 2Ar-H, *J *= 8.09 Hz), 7.43 (t, 1Ar-H, *J *= 7.43 Hz), 7.01 (d, 2Ar-H, *J *= 8.60 Hz), 5.63 (s, 2H, C*H_2_*COOH), 3.81 (s, 3H, OCH_3_). ^13^C NMR (DMSO-d_6_, 100 MHz, δ ppm): 166.5 (COOH), 158.6 (tetrazole carbon), 149.5, 138.5, 135.1, 129.7, 128.3, 127.5, 126.0, 124.5, 118.4, 113.5, 107.5, 54.5 (CH_2_), 53.5 (OCH_3_). MS (m/z): 376 (M^+^). 

#### 2-(5-(3-(2-naphthyl)-1-phenyl-1H-pyrazol-4-yl)-2H-tetrazol-2-yl) acetic acid (5g)

Yellow powder, yield 62 %, mp 210-212 °C. IR (KBr, cm^-1^): 3498 (OH), 3051 (CH aromatic), 1720 (C=O), 1593 (C=N). ^1^H NMR (DMSO-*d*_6_, 400 MHz, δ ppm): 10.84 (s, 1H, COOH), 9.04 (s, 1H, pyrazole), 8.22 (d, 2Ar-H, *J *= 8.0 Hz), 8.00-7.94 (m, 3Ar-H), 7.71-7.65 (m, 2Ar-H), 7.60-7.55 (m, 4Ar-H), 7.40 (t, 1Ar-H, *J *= 7.23 Hz), 5.82 (s, 2H, C*H_2_*COOH). ^13^C NMR (DMSO-d_6_, 100 MHz, δ ppm): 167.8 (COOH), 157.9 (tetrazole carbon), 140.2, 137.6, 132.4, 131.4, 129.4, 128.5, 127.8, 126.6, 126.4, 125.8, 124.2, 123.4, 118.6, 103.7, 52.7 (CH_2_). MS (m/z): 396 (M^+^). 

### Pharmacological screening 

#### In vitro antiproliferative activity

The antiproliferative activity of synthesized compounds was evaluated against HT-29 colon cancer and PC-3 prostate cancer cell lines as described in the literature (Lembege et al., 2008[18]; Chen et al., 2015[7]). Tumor cell lines were grown in DMEM media supplemented with 20 % heat-inactivated fetal bovine serum (FBS), 100 IU/mL penicillin, 100 mg/mL streptomycin and 2 mM-Glutamine. The cell cultures were maintained in a humidified atmosphere with 5 % CO_2_ at 37 °C. The cells were subcultured twice a week, seeding at a density of about 2×10^3^ cells/mL. Cell viability was determined by the trypan blue dye exclusion method. 5×10^3^ cells/well (HT-29 and PC-3) were inoculated in 96-well microtiter plate for 24 h, before treatment with the compounds, cells were allowed to attachment with wall of the plate. Test compounds were dissolved in dimethyl sulfoxide (DMSO), further diluted with saline to appropriate volume. Different concentrations of the compound under test (0.5, 1, 5, and 10 μM/mL) were added to the wells, and the cells were incubated at 37 °C in a 5 % CO_2 _incubator for 48 h. The cells were treated with 10 μL MTT dye solution (5 mg/mL) for 4 h cultivation. The media along with the MTT solution was washed with 100 μL of DMSO solution. The absorbance of formazan solution was measured at 540 nm with an automatic multi-well plate reader (Victor3^TM^, Perkin-Elmer, and USA). Percentage inhibition of proliferation was calculated as a fraction of control (without drug) 

%Growth = Absorbance of test/Absorbance of control×100

% Inhibition = 100- % Growth

#### Angioinhibitory activity by CAM assay

CAM assay was performed according to the previously described method (Chandru et al., 2007[6]). The fertilized eggs were divided into sixteen groups and in each group a minimum of six eggs were used. Vehicle control group was treated with 0.1 % DMSO, saline treated group and compounds treated groups were maintained separately and observed. For a period of 5 days, eggs in all the groups were incubated separately at 37 °C in humidified and sterile conditions. A small window was made on egg shell under aseptic conditions and observed for proper development of the embryo. The windows were resealed with adhesive tape and incubation was continued. On day 11, the windows were opened and the test compounds and vehicle were loaded on the cover slips separately, air-dried, and inverted over the CAM. The windows were resealed and returned to the incubator. The windows were opened and inspected on day 13 and observed for changes of MVD in the area under cover slip and studied under a microscope for avascular zone.

#### In vivo antitumor activity

The experimental protocol for the pharmacological screening on mice was done with an Institutional Animal Ethics Committee, Guru Nanak Institute of Pharmacy, Hyderabad, India (Reg no: 1374/ac/10/ CPCSEA). Male Albino Swiss mice weighing between 20-25 g were selected for screening of anticancer activity. Animals were kept and maintained under standard laboratory conditions and provided free access to food and water ad libitum. The selected animals were allowed to acclimatize to the laboratory environment for seven days prior to *in vivo* experiment. The selected animals were divided into 9 groups each comprised of 12 animals. EAC cells were obtained from the donor mice and were suspended in a known quantity of 0.9 % NaCl normal saline. The cell count was adjusted to 2×10^6^ cells/mL. All the groups were treated with EAC cells through intraperitonial (ip) route except the normal control group on day zero. Group I was treated with normal saline (5 mL/ kg body weight) and group II as EAC control. Group III was treated with standard drug 5-Fluorouracil (20 mg/kg body weight). The test compounds were administered through ip at dose of 50 mg/kg body weight in groups IV-IX, respectively. All the test compounds and 5-Fluorouracil were treated daily for 9 days starting 1 day after tumor transplantation. After 9 days, six animals from each group were sacrificed. The tumor volume and cell count parameters were recorded. Mean survival time (MST) for remaining six mice of each group was recorded (Dhamija et al., 2013[10]).

The groups and design of experiment:

Group I: Normal saline

Group II: EAC (2×10^6^ cells)

Group III: EAC (2×10^6^ cells) + 5-Fluorouracil

Group IV: EAC (2×10^6^ cells) + Compound 4b 

Group IV: EAC (2×10^6^ cells) + Compound 4c 

Group V: EAC (2×10^6^ cells) + Compound 4f 

Group VI: EAC (2×10^6^ cells) + Compound 5b 

Group VII: EAC (2×10^6^ cells) + Compound 5c 

Group VIII: EAC (2×10^6^ cells) + Compound 5f 

#### Tumor weight and cell count

The 6 mice were dissected and the total ascetic fluid was harvested from peritoneal cavity. The volume was measured by taking it in a graduated centrifuge tube and the packed cell volume noted by centrifuge at 1000 rpm for 5 min and viable cells were checked by trypan blue dye exclusion test and cells were counted in Neubauer counting chamber.

MST and percentage increase in life span ( %ILS)

The activity of the pyrazole compounds on tumor growth was evaluated by recording the mortality within the observation period. Percentage increase in life span (ILS) was calculated by using the following formula.

% ILS = (MST of treated group - MST of control group ×100) / MST of control group

MST = (Survival time of each mice in a group (days)) / Total number of mice

#### Molecular modeling 

Docking was performed on windows 2002 using MOE 2008.10 version. VEGFR-2 kinase was retrieved from the protein data bank (PDB code: 2XIR) and the receptor was visualized using sequence option and further co-factors were deleted. The partial charge of protein was adjusted with the help of force field method AMBER 99. Later, the protein was subjected to 3D protonation at cut off 12.0, and further hydrogen was added according to standard geometry and the receptor was energy minimized using force field MMFF94x at 0.01 KJ mole gradients. The ligand structures were written by using a builder module, and adjusting the partial charges using Hamilton MMFF94 force field method and subsequently 3D protonation and hydrogen addition was performed according to standard geometry. Ligands were energy minimized at cut off 12 using force field MMFF94x at 0.01KJ mole gradient. Docking was performed using the option simulation followed by dock on selected active site amino acids using sequence option, and eventually docked using setting options such as receptor and solvent, alpha triangle, selected residues, affinity dG, force field refinement and best 30 pose. After obtaining docking results, out of the 30 best posed results for each chemical structure, best pose was retained. The resultant best pose score values in the series were used for analysis of docking and interaction (Garofalo et al., 2011[13]). 

#### Toxicity prediction by bioclipse 

Bioclipse open tox software was run on windows 7 platform, which process the chemical structure by interacting with known toxic compounds for predictive toxicology. Chemical structures were imported in mole file into bioclipse navigator which was further processed with different interacting software modules such as mutagenicity, carcinogenicity, aquatic toxicity, site of metabolism and Cytochrome P450 proteochemometric (CYP PCM). After ballooning, obtained detailed decision support in portable document format (Spjuth et al., 2011[30], 2007[31]).

#### Assessment of lipophilicity and drug score profiles 

Shredding of each molecule at every rotatable bond led to a set of core fragments. These in turn were used to reconstruct all possible larger fragments which could be the substructure of the original molecule. Afterward, a search process of substructure determined the occurrence frequency of every one of the fragment (constructed and core fragments) within all traded dugs of 3300 as well as 15,000 commercially available chemicals (Fluka) to predict lipophilicity and drug score profiles (Ali et al., 2013[1]).

## Results and Discussion

### Chemistry

According to Figure 1(Fig. 1), pyrazole-4-carbaldehyde **1** was reacted with hydroxylamine hydrochloride to give corresponding pyrazolyl oxime **2** by established reaction. The resultant product was confirmed by appearance of a peak at 1620 cm^-1^ and disappearance of aldehyde peak at 1670 cm^-1 ^in IR spectra. The pyrazolyl oxime was heated with acetic anhydride at 140-150 °C for 2 h to give pyrazole nitrile **3**, which was confirmed by a sharp peak around 2200 cm^-1 ^in IR spectra. The compound **3** was cyclized with sodium azide in the presence of triethyl ammonium chloride under reflux for 24-36 h to yield **4a-4g. **The formation of these compounds was confirmed by disappearance of peak at 2200 cm^-1^ and appearance of NH peak at 3400-3420 cm^-1 ^in IR spectra.^ 1^H NMR spectra of compounds showed a singlet around δ 9.02 for pyrazole proton and the peaks for aromatic protons appeared in the range of δ 7.00-8.00 validated formation of desired compounds. The compounds **4a-4g **were treated with ethyl chloroacetate yielded corresponding esters, which were hydrolyzed with sodium hydroxide to give desired compounds **5a-5g**. The structures of final compounds were confirmed using FTIR,^ 1^H NMR,^ 13^C NMR, and mass spectral data. In IR spectra, C=O absorption band of COOH appeared between 1720- 1730 cm^-1^ and ^1^H NMR of compounds showed a singlet between δ 10.50 to11.00 for COOH proton, a singlet around δ 9.00 for pyrazole proton, a singlet at δ 5.50-5.80 for two methylene protons of CH_2_COOH and the peaks for aromatic protons appeared in the range of δ 7.00-8.50 confirmed the structures. ^13^C NMR spectra of compounds showed a peak in the range of δ 47.0-54.0 due to CH_2_ carbon, the carbon of COOH appeared around δ 167.0, a peak around δ 157.0 due to tetrazole carbon and aromatic carbons appeared in the range of δ 104.0-150.0 confirmed the formation of desired compounds. This was further more supported by mass spectral data. The physical and spectral data of individual compounds were given under experimental section.

### Pharmacological screening 

#### In vitro antiproliferative activity

Antiproliferative screening of the synthesized compounds **4a-4g** and** 5a-5g** was carried out by *in vitro* method using MTT assay against two cell lines namely HT-29 (colon) and PC-3 (prostate). Doxorubicin was used as reference drug. From percentage inhibition IC_50 _values were calculated in µM and shown in Table 1(Tab. 1). Overall, better antiproliferative activity was observed for **5a-5g** series as compared to **4a-4g** series against two cell lines. Particularly, compounds possessing chloro and methoxy substitutions on phenyl ring at para position **(5c **and** 5f**) showed highest antiproliferative activity against HT-29 and PC-3 cell lines with IC_50_ values ranging from 6.43 µM to 10.15 µM as compared to corresponding ortho substituted compounds (**5b and 5e**). However, compounds with methyl substitution at para position (**4d **and** 5d**), compounds **4a** and **5a,** and napthyl derivatives (**4g** and **5g**) exhibited less antiproliferative activity. 

#### Evaluation of angioinhibitory activity by CAM Assay 

**Figure 2:**
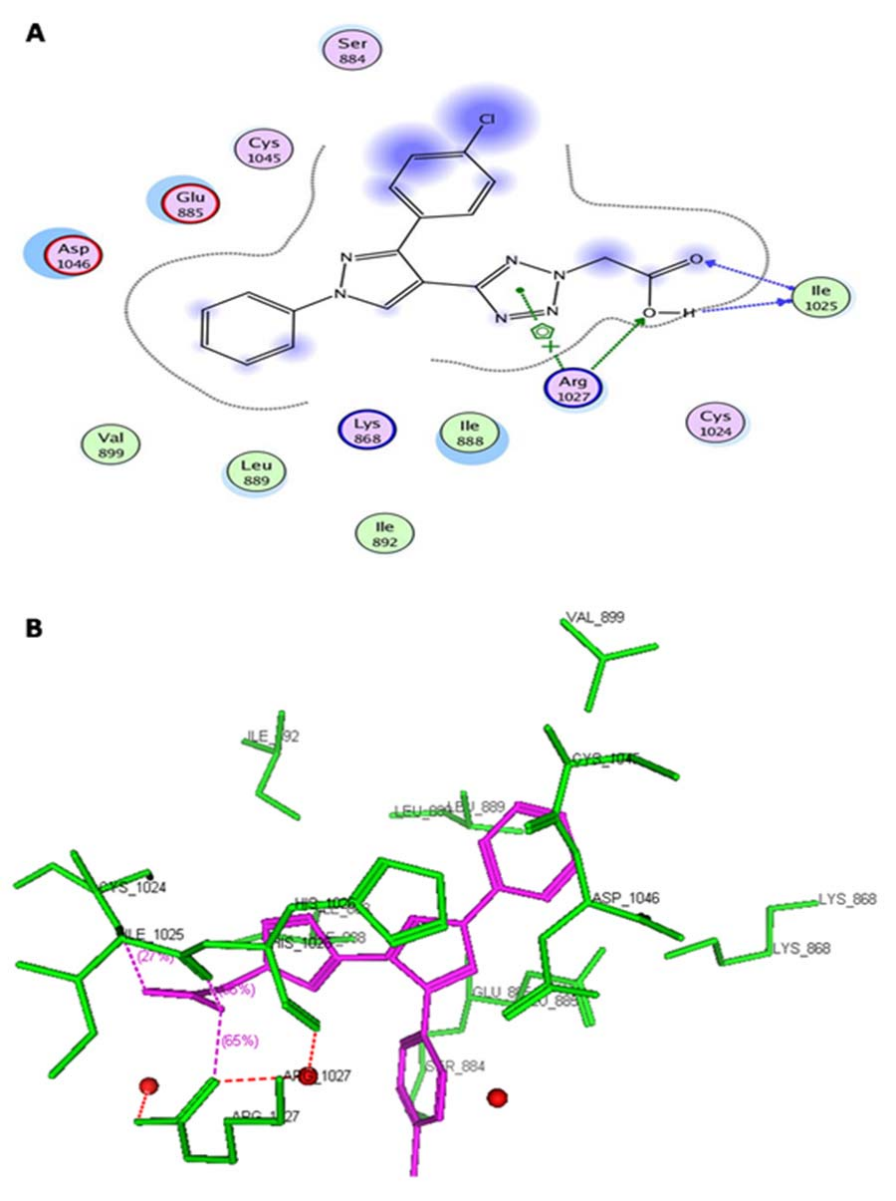
(A) Two-dimensional representation of the interacting mode of 5c with VEGFR-2 kinase. (B) Three-dimensional structural model of compound 5c (purple) into VEGFR-2 kinase

The antiangiogenic activity was screened by observing microvessel density (MVD) count, which is important tool to assess the neovasculature in tumors. Vehicle control group was treated with 0.1 % DMSO did not show any change in vasculature. CAM assay results showed reduced MVD count for the synthesized pyrazole derivatives. There is remarkable correlation observed between the docking studies and angioinhibitory activity. Among the tested compounds, compound **5c **exhibited the lowest MVD count showed significant docking interactions with the VEGFR-2 active site (Figure 2(Fig. 2)). 

#### Evaluation of in vivo antitumor activity against EAC 

Based on promising results obtained in early assays (*in vitro* and CAM), compounds were selected namely **4b, 4c, 4f, 5b, 5c** and **5f **for *in vivo* anticancer against EAC bearing mice. The reduction in viable tumor cell count, MST, and %ILS are important measures that have been used in this *in vivo* testing. 5-Flurouracil (20 mg/kg body weight) was used as standard drug. Obtained results were shown in Table 2(Tab. 2).

#### Effect on survival time

All *in vivo* tested compounds showed significant increase in MST and % ILS as compared to EAC control (Table 2(Tab. 2)). Compound **5c **exhibited higher antitumor activity and showed 32.4 ± 0.53 days of MST with 75.13 % ILS as compared to rest of the compounds in the series (**5a-5g** and **4a-4g**). Compound **5f **showed better 30.3 ± 1.58 days of MST with 63.78 % ILS as compared to compound **5b **(28.6 ± 1.23 days of MST with 54.59 % ILS). Whereas, compounds **4f (**27.0 ± 1.25 days of MST with 45.94 % ILS), and **4c (**26.2 ± 1.21 days of MST with 41.62 % ILS) displayed moderate activity.

#### Tumor volume and viable cells

Antitumor activity of tested compounds on EAC bearing mice *in vivo* was further studied in terms of tumor volume and number of viable cells. Almost, all the tested compounds exhibited significant decrease in tumor volume and cell number as compared to EAC control. Compound **5c, **which exhibited highest, % ILS, demonstrated maximum reduction of viable cells (33.5 ± 1.6 %) and tumor volume (1.7 ± 0.03 mL) as compared to rest of the compounds.

### Docking studies 

**Figure 3:**
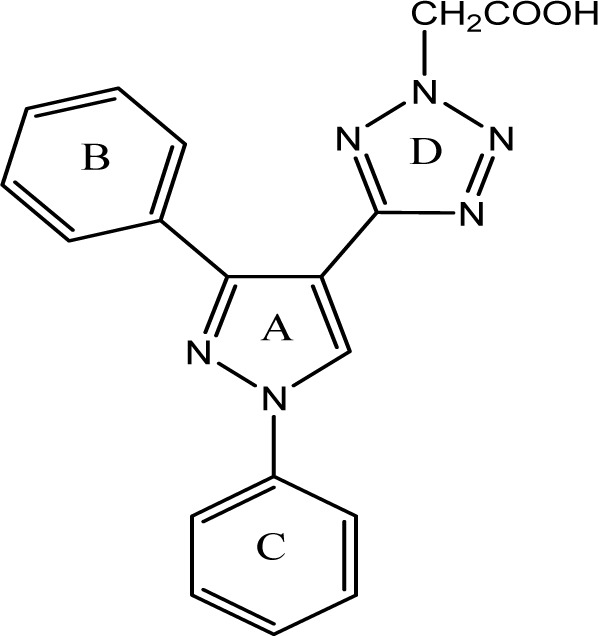
General structure of designed pyrazole derivatives

**Figure 4:**
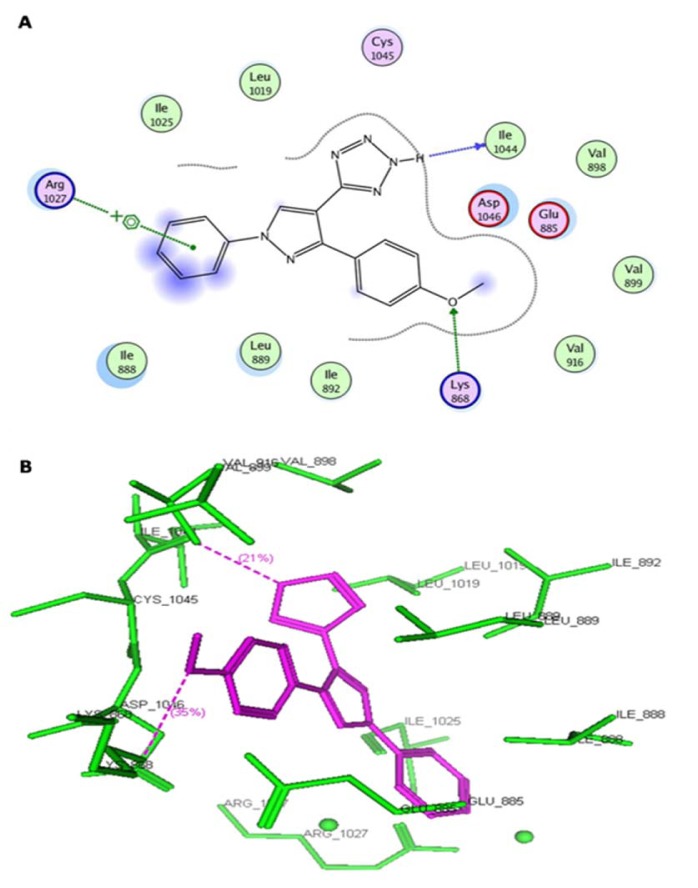
(A) Two-dimensional representation of the interacting mode of 4f with VEGFR-2 kinase. (B) Three-dimensional structural model of compound 4f (purple) into VEGFR-2 kinase

Virtual pyrazole derivatives (Figure 3(Fig. 3)) showing significant interactions in molecular docking, this provided guidance for selection of derivatives to be synthesized for better antiproliferative activity. Literature survey reveals that, VEGFR-2 kinase inhibitors known to interact with amino acids such as Asp 1046, Glu 885, Cys 1045, Val 895, Arg 1027, Phe 1047, Ile 1025, etc., at the active site. While Asp 1046, Glu 885, and Arg 1027 tend to form hydrogen bonds with the ligand, Phe 1047 and Val 899 undergo stacking interactions with aromatic ring of ligand (Meenakshi et al., 2013[23]; Sanphanya et al., 2013[29]; Borzilleri et al., 2005[4]; Dai et al., 2008[9]). In the present study, the designed structures were docked into VEGFR-2 kinase active site to explore to know that experimental compounds have similar interactions as that of known VEGFR-2 inhibitors. Docking of compound **4f** showed two hydrogen bond interactions (NH of ring D and Ile 1044; d=1.88 A^o^, OCH_3_ of ring B and Lys 868; d=3.06 A^o^) and ring C showed stacking interaction with Arg 1027 (d=3.64 A^o^). Moreover, ring B was surrounded by Asp 1046 and Glu 885 and this may be the reason for superior activity of compound **4f** among the series **4a-4g** in antiproliferative studies. Compound **4e**, though it does not show any hydrogen bond interactions in docking, but elicited moderate antiproliferative activity studies, which might be due to its close association with active site amino acids such as Asp 1046, Lys 868, His 1026 and Glu 885. Compound **5c** exhibited three hydrogen bond interactions (oxygen of OH and Arg 1027; d=2.28 A^o^, hydrogen of OH and Ile 1025; d=1.23 A^o^, C=O and Ile 1025; d=2.89 A^o^) and ring D stacking interaction with Arg 1027. Apart from this, compound was surrounded by Asp 1046, Glu 885 and Lys 868. The introduction of -CH_2_COOH in the series **5a-5g** provided additional hydrogen bond interactions with the active site amino acids of VEGFR-2 and this might have contributed for better activity of these series as compared to other series of compounds **4a-4g, **which is supported by results obtained in antiangiogenesis, *in vitro* and *in vivo* antiproliferative activity**.** Data pertaining to the interaction of pyrazolyl tetrazoles **4a-4g** and pyrazolyl tetrazole acetic acids **5a-5g** with active site amino acids of VEGFR-2 was given in Table 3(Tab. 3). The two-dimensional and three-dimensional representations of compound** 5c** and **4f **were given under Figures 2(Fig. 2) and 4(Fig. 4).

### Structure activity relationship study (SAR) 

Structure activity relationship was carried out according to the results of *in vitro* and *in vivo* antiproliferative activity of the screened compounds. It is interesting to note that **5a-5g** series have more antiproliferative activity as compared to that of **4a-4g** series against tested cell lines and EAC bearing mice. Particularly, compound **5c **possessing para chloro on phenyl ring showed more potent activity against two cell lines HT-29 and PC-3, and EAC *in vivo* as compared to ortho chloro substitution on phenyl ring (**5b**). However, para methoxy group on the phenyl ring (**5f**) demonstrated better *in vitro* activity as compared to ortho methoxy derivatives (**5e**). In the series of the pyrazole tetrazolyl derivatives, para methoxy substitution on the phenyl ring (**4f**) exhibited significant *in vitro* and moderate *in vivo* antiproliferative activity. Moreover, chloro substitution at para and ortho positions (**4c** and **4b**) showed slightly less activity as compared to (**4f**). Methyl substitution at para position (**4d **and** 5d**), compounds with unsubstituted (**4a** and **5a),** and napthyl derivatives (**4g** and **5g**) showed less antiproliferative activity. 

### Toxicity prediction by bioclipse

Bioclipse online software was used for the prediction of the overall toxicity of the new compounds before they are being synthesized and screened for activity. The prediction strategies rely on efficient mapping of complementary data coming from different data set into a unifying structure having shared terminology and representation. Mutagenicity is the ability of a substance to induce mutations by interacting with DNA and to change its structure. A carcinogen is a type of mutagen that specifically causes cancer and aquatic toxicology parameter is meant to study the effects of manufactured chemicals on aquatic organisms. 

Site of metabolism prediction process focuses on pinpointing the site or moiety in a chemical structure, which is most likely to undergo metabolization, hence aiding with decision support in the drug optimization process. A positive result implies susceptibility of the moiety for metabolism. Negative results indicate the resistance of the moiety for undergoing metabolism. Cytochrome P450 (CYP) isoforms are responsible for metabolism of many drugs. Inhibition of this CYP isoforms results in decreased elimination and change in metabolic pathways of their substrates, which is the major cause of adverse drug-drug interactions. It is, therefore, essential to trace out the compounds potential for CYP inhibition at an early stage of drug discovery. The ability of the designed compounds to inhibit five major drug metabolizing CYP isoforms (i.e. CYP3A4, CYP2D6, CYP2C19, CYP2C9 and CYP1A2) was predicted by using a unified proteochemometric (PCM) model in bioclipse software. A positive result implies that compound computationally predicted to inhibit CYP isoforms and the negative sign indicates that the compound computationally predicted to have no ability to inhibit CYP isoforms. The designed pyrazolyl tetrazoles **4a-4g **and **5a-5g** have shown differential effect on the inhibition of CYP isoforms. While compounds **4a-4c** predicted to show positive on the inhibition of CYP isoforms and the remaining compounds exhibited negative effect. It can be assumed that the presence of -CH_2_COOH moiety in **5a-5g** series may be responsible for the negative effect on inhibition of CYP isoforms. The results displayed in Table 4(Tab. 4), showed that all the compounds are likely to exhibit low probable toxicity risks as revealed by computational *in silico* studies.

### Assessment of lipophilicity and drug score profiles

Osiris program was used for prediction of the C log P of the designed derivatives. C Log P is a well-established parameter to measure the compound hydrophilicity. Compounds show reasonable probability of being well absorbed, when they have C log P value less than 5.0. It is well established that 80 % of the drugs on the market have log S value around -4.0. From the table, it was observed that all designed compounds have shown C log P less than 5.0 and most of the compounds have log S values around -4.0 indicating that the synthesized compounds could be possible drug candidates. The drug score combines C Log P, Log S, molecular weight and toxicity risks in one handy value that may be used to judge the compounds overall potential to qualify for a drug. A value around 0.5 makes this compound a promising lead for future development of safe and efficient drug. Predictions of C log P, solubility, and drug score for the title compounds are given in Table 5(Tab. 5) and almost all the designed compounds possess good values of drug score. 

## Conclusion

A novel series of pyrazolyl tetrazoles **4a-4g** and pyrazolyl tetrazole acetic acids **5a-5g** were designed, synthesized and evaluated for *in vitro* antiproliferative activity against two cell lines and angioinhibitory activity in CAM assay. Based on the obtained results of *in vitro* and CAM assays, compounds were selected for *in vivo* studies. Among the tested derivatives, compound possessing para chloro substitution on phenyl ring **(5c**), which showed significant activity *in vitro* and *in vivo* potency, also exhibited better angioinhibitory activity. Assessment of toxicities and drug score profiles of designed compounds are promising. Moreover, the synthesized compounds showed significant docking interactions with VEGFR-2 active site. All the results showed that the tested compounds may lead to discovery of potent anticancer agents for further optimization.

## Acknowledgements

This project is financially supported by University Grants Commission, New Delhi, India (MRP 6254/15/SERO/UGC).

## Conflict of interest

The authors declare no conflict of interest.

## Figures and Tables

**Table 1:**
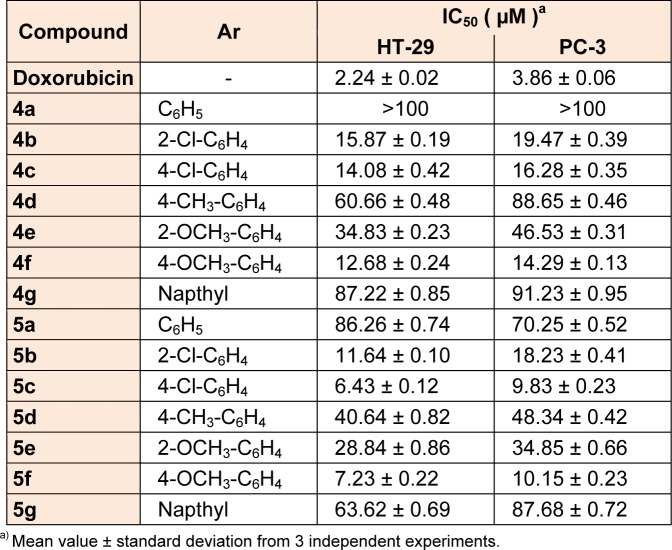
*In vitro* antiproliferative activity of synthesized compounds against two cell lines

**Table 2:**
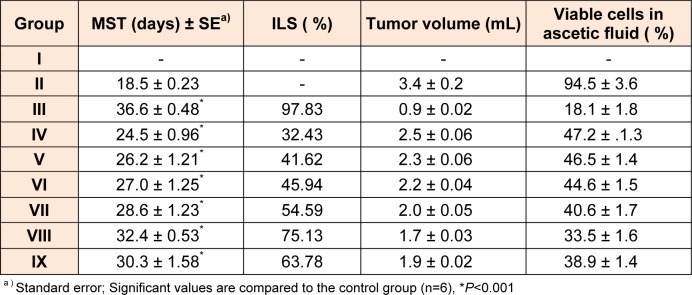
*In vivo* antiproliferative activity of 4b, 4c, 4f, 5b, 5c and 5f against EAC bearing mice

**Table 3:**
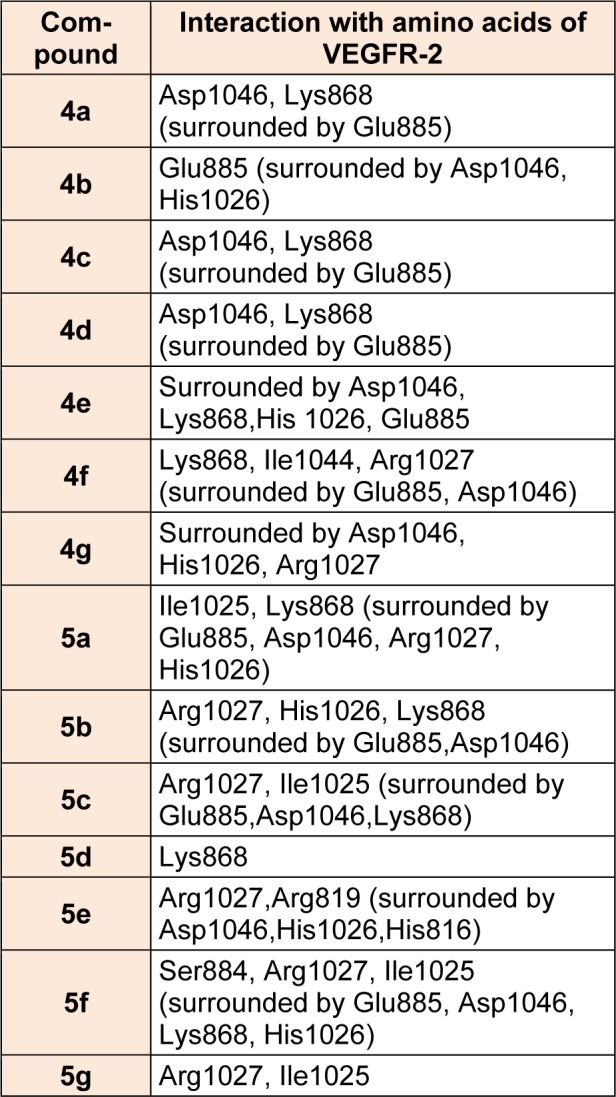
Interaction of pyrazolyl tetrazoles 4a-4g and pyrazolyl tetrazole acetic acids 5a-5g with active site amino acids of VEGFR-2 receptor

**Table 4:**
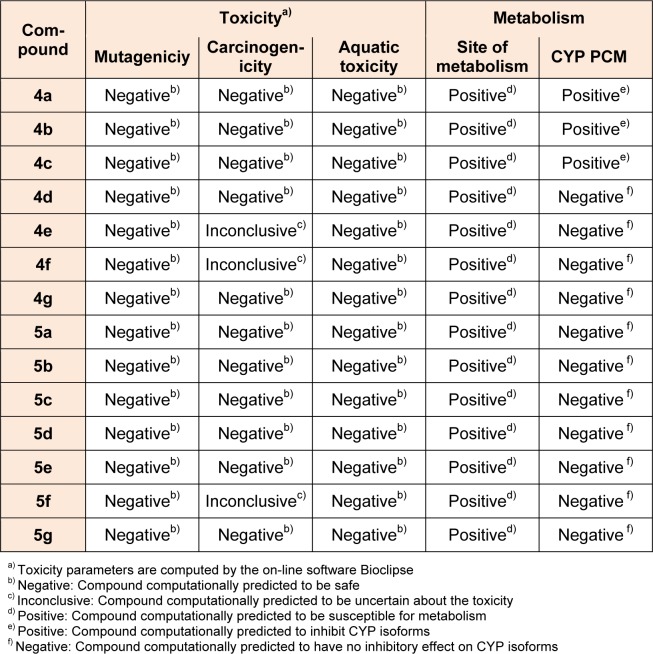
Predicted toxicity parameters of synthesized 4a-4g and 5a-5g compounds

**Table 5:**
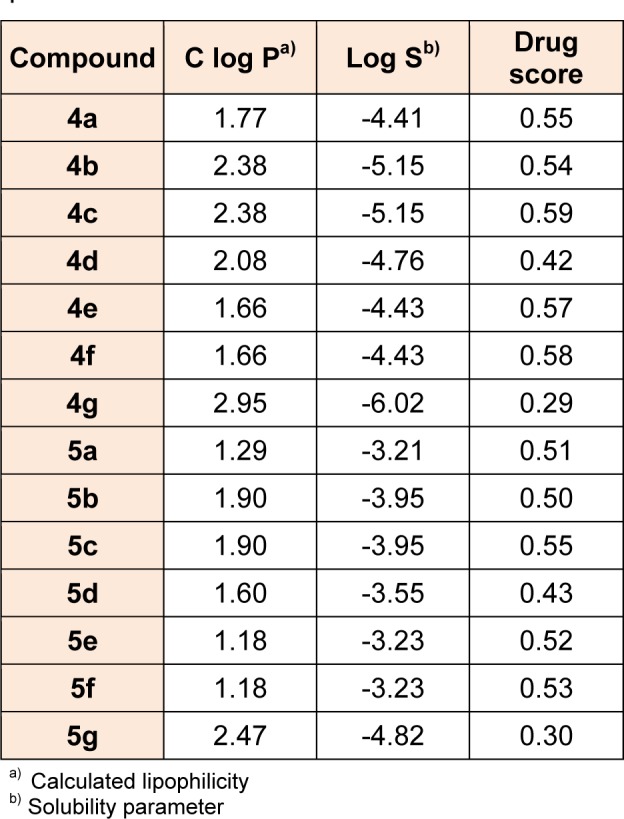
Computationally predicted lipophilicity, solubility and drug score of the designed compounds
